# Breastfeeding Status and Duration and Infections, Hospitalizations for Infections, and Antibiotic Use in the First Two Years of Life in the ELFE Cohort

**DOI:** 10.3390/nu11071607

**Published:** 2019-07-15

**Authors:** Camille Davisse-Paturet, Karine Adel-Patient, Amandine Divaret-Chauveau, Juliette Pierson, Sandrine Lioret, Marie Cheminat, Marie-Noëlle Dufourg, Marie-Aline Charles, Blandine de Lauzon-Guillain

**Affiliations:** 1 Université de Paris, CRESS, INSERM, INRA F-75004 Paris, France; 2 UMR Service de Pharmacologie et Immunoanalyse, CEA, INRA, Université Paris-Saclay, 91191 Gif-sur-Yvette, France; 3 Unité d’allergologie pédiatrique, Hôpital d’enfants, CHRU de Nancy, 54500 Vandoeuvre-lès-Nancy, France; 4 EA3450, DevAH-Department of Physiology, Faculty of Medicine, University of Lorraine, 54500 Vandoeuvre-lès-Nancy, France; 5 Ined, Inserm, Joint Unit Elfe F-75020 Paris, France

**Keywords:** breastfeeding, infections, birth cohort, hospitalizations, antibiotic use

## Abstract

In low- and middle-income countries, the protective effect of breastfeeding against infections is well established, but in high-income countries, the effect could be weakened by higher hygienic conditions. We aimed to examine the association between breastfeeding and infections in the first 2 years of life, in a high-income country with relatively short breastfeeding duration. Among 10,349 young children from the nationwide Etude Longitudinale Française depuis l’Enfance (ELFE) birth cohort, breastfeeding and parent-reported hospitalizations, bronchiolitis and otitis events, and antibiotic use were prospectively collected up to 2 years. Never-breastfed infants were used as reference group. Any breastfeeding for <3 months was associated with higher risks of hospitalizations from gastrointestinal infections or fever. Predominant breastfeeding for <1 month was associated with higher risk of a single hospital admission while predominant breastfeeding for ≥3 months was associated with a lower risk of long duration (≥4 nights) of hospitalization. Ever breastfeeding was associated with lower risk of antibiotic use. This study confirmed the well-known associations between breastfeeding and hospitalizations but also highlighted a strong inverse association between breastfeeding and antibiotic use. Although we cannot infer causality from this observational study, this finding is worth highlighting in a context of rising concern regarding antibiotic resistance.

## 1. Introduction

In 2013, infectious diseases were in the four main categories of leading causes of death among children under 5 years of age worldwide [1]. Nonetheless, disparities exist between countries. Infections are the leading causes of death in sub-Saharan African countries but not in high-income countries. The good hygienic conditions and health care system available in some countries significantly reduce the prevalence and fatal issue of such diseases but do not fully prevent them.

The World Health Organization (WHO) recommends exclusive breastfeeding for 6 months, or at least the first 4 months of life [2]. These recommendations were mainly based on the protective effect of breastfeeding against infectious morbidity and mortality [3]. In fact, breast milk components, such as immunoglobulin A (IgA) or maternal leukocytes, can both supplement and promote the newborn’s immature immune system [4] and therefore lead to protective effect against infections.

More precisely, recent literature has shown that breastfeeding is related to a reduced rate of hospital admission for diarrhea and respiratory infections as well as a protective effect on otitis media in children up to 2 years old [3,5]. Of note, otitis media studies were mostly from high-income countries, whereas results on diarrhea and respiratory infection studies were mostly found in settings from low- and middle-income countries [6,7]. In high-income countries, the preventive effect of breastfeeding on respiratory tract infections is less consistent across studies [7]. In the cluster-randomized trial on promotion of breastfeeding (PROBIT), which took place in Belarus in the 1990s, breastfeeding was related to a reduced risk of gastrointestinal infections in the first year of life [8].

The aim of this study was to assess the association between breastfeeding duration and several indicators of infectious morbidity, in France, a high-income country with the specificity of low breastfeeding initiation rate (69.7%) and median duration below the guidelines (17 weeks among breastfeeding mothers) [3,9].

## 2. Materials and Methods

### 2.1. Study Population

This analysis was based on data from the ELFE (Etude Longitudinale Française depuis l’Enfance) study, a multidisciplinary nationwide birth cohort including 18,329 children born in 2011 in France [10]. The inclusion criteria were as follows: singleton or twins born after 33 weeks of gestation, to mothers aged 18 years or older. Participating mothers had to provide written consent for their own and their child’s participation. Fathers signed the consent form for the child’s participation when present at inclusion or were informed about their rights to oppose it. The ELFE study was approved by the Advisory Committee for Treatment of Health Research Information (Comité Consultatif sur le Traitement des Informations pour la Recherche en Santé), the National Data Protection Authority (Commission Nationale Informatique et Libertés), and the National Statistics Council.

### 2.2. Breastfeeding

The feeding method was prospectively collected up to 2 years and the calculation of breastfeeding duration was detailed in a previous paper [11]. As previously described, two breastfeeding definitions were used in the present study: any breastfeeding and predominant breastfeeding. Any breastfeeding was defined as the infant receiving breastmilk. Predominant breastfeeding was defined as the only milk given to the infant being human milk (no animal milk or infant formula).

For each breastfeeding definition, infants were first categorized as ever or never breastfed and then according to their breastfeeding duration (never, <1 month, 1 month to <3 months, 3 months to <6 months, ≥6 months).

### 2.3. Parental Report of Infections

In the present study, infections were assessed with several indicators: hospitalizations; frequency of bronchiolitis and otitis events; and antibiotic use.

At the 1- and 2-year phone interviews, parents reported hospitalizations in the previous year (date, duration, and main cause). Infants with at least one hospitalization for infectious disease were identified (mainly fever, gastrointestinal infection, and bronchiolitis). Hospitalizations from infectious diseases between birth and 2 years of age were characterized by the number of events (none, 1, ≥2) and cumulative duration (never, 1–3 nights, ≥4 nights).

At the 1-year phone interview, parents both reported whether the child ever had a bronchiolitis event and if the child had had at least 3 bronchiolitis events since birth. At the 2-year phone interview, parents reported whether the child had had 3 bronchiolitis episodes or more since birth. A mixed variable was computed from both interviews resulting in a 3-category variable assessing bronchiolitis events in the first 2 years of life (never, 1 or 2 events, ≥3 events).

At the 2-year phone interview, parents reported otitis events since birth (<3 events, ≥3 events). Unfortunately, it was not possible to distinguish infant with no otitis event from those having 1 or 2 events.

At the 1- and 2-year phone interviews, parents reported the frequency of antibiotic use for their child in the previous 12 months. These frequencies were then combined into a 4-category variable (never, once, 2 or 3 times, >3 times).

### 2.4. Other Variables

Maternal and household data were collected using face-to-face interviews at the maternity unit and then by phone interview at the 2-month follow-up. Because these data were more thoroughly assessed during the 2-month interview and only marginally changed during these 2 months, we used the data collected at 2 months in our analyses. Socio-demographic characteristics collected during the maternity stay were used only when the 2-month values were missing.

Maternal socio-demographic characteristics included age at the birth of her first child (<25, 25–29, 30–34, ≥35 years), education level (below secondary school, secondary school, high school, 2-year university degree, 3-year university degree, 5-year university degree or higher), place of birth (France, abroad), and employment status (unemployed, employed). Household characteristics included income per consumption unit (≤€750, €751 to 1111, €1112 to 1500, €1501 to 1944, €1945 to 2500, >€2500 /month) and composition (couple with children, single parenthood, step family).

Maternal health-related characteristics included smoking status during pregnancy (never smoked, smoked only before pregnancy, smoked only in early pregnancy, smoked throughout pregnancy) and pre-pregnancy body mass index (BMI) (<18.5 kg/m², 18.5 to 24.9 kg/m², 25.0 to 29.9 kg/m ², ≥30.0 kg/m²).

Infant’s birth order (first born, second, third, fourth, or higher), caesarean-section delivery, sex, twin birth, and gestational age were collected at birth from medical records. Infant’s age at first attendance at a shared childcare facility was computed from the 1-year phone interview as a 5-category variable (≤2 months, >2 to ≤4 months, >4 to ≤6 months, >6 to ≤12 months, never attended in the first year).

### 2.5. Sample Selection

Infants whose parents withdrew consent during the first year (*n* = 57) and not meeting eligibility criteria (*n* = 1) were excluded, resulting in 17,984 eligible infants. In twin pregnancies, one twin was randomly selected (*n* = 287 exclusions) to avoid family clusters.

**Figure 1:**
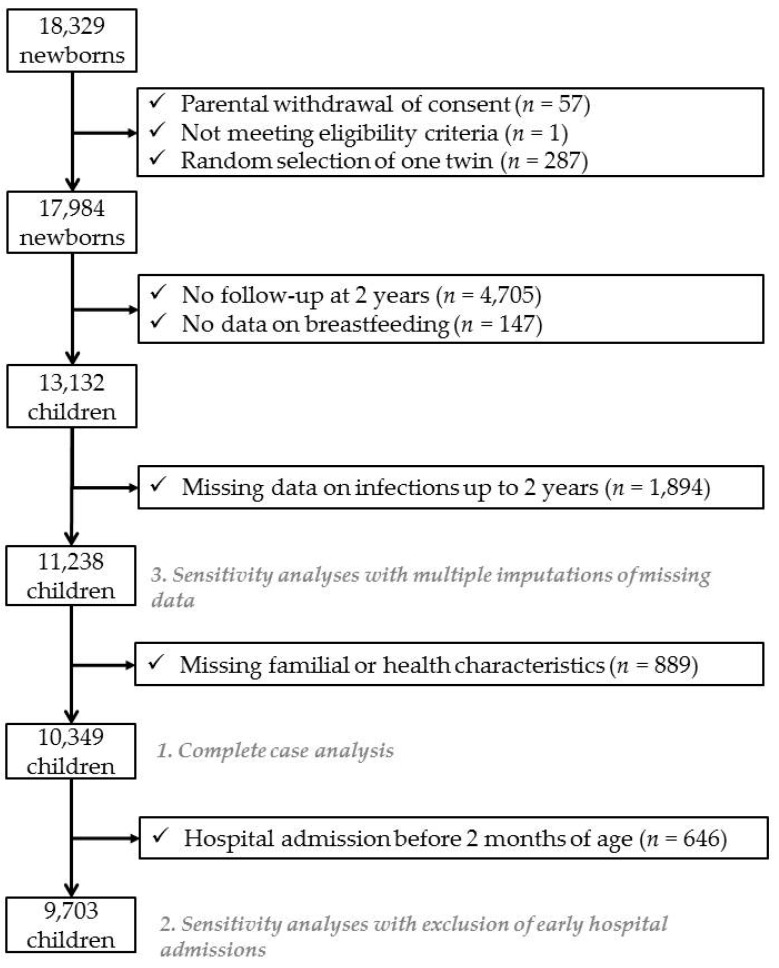
Sample selection.

We excluded infants without any follow-up at 2 years (*n* = 4705). We then excluded infants with missing data on breastfeeding (*n* = 147) and those with missing data on infections or antibiotic use (*n* = 1894). We also excluded infants with incomplete information for potential confounding variables (*n* = 889). These exclusions lead to a sample of 10,349 infants for the complete case analysis regarding parental reports of infections and antibiotic use (Figure 1).

### 2.6. Analyses

#### 2.6.1. Main Analyses

To compare selected families to their non-selected ELFE counterparts, we used chi-squared tests for categorical variables and Student *t*-tests for continuous variables.

Associations between breastfeeding and parental reports of infections or antibiotic use were assessed with multinomial logistic regression models. Never-breastfed infants were systematically used as the reference group. In all analyses, we adjusted for potential confounding factors (maternal age at first child, education level, employment status, smoking status during pregnancy and pre-pregnancy BMI, household monthly income per consumption unit, household composition, caesarean section and infant’s sex, gestational age, birth order, and age at first attendance at a shared childcare facility), and variables related to study design (recruitment wave, maternity unit size, and mother’s region of residence).

#### 2.6.2. Sensitivity Analyses

As early hospitalizations may lead to early breastfeeding cessation, we repeated the main analyses after excluding infants with hospitalizations occurring before the age of 2 months, leading to a sample of 9703 infants.

To deal with missing data for potential confounding factors, we first conducted our analyses on complete cases (Figure 1, *n* = 10,349). Secondly, we used multiple imputations to deal with these missing data. This method assigned data to missing measurements based on the measurement of infants with similar profiles. We assumed that data were missing at random and generated five independent datasets with the fully conditional specification method (MI procedure, FCS statement, NIMPUTE option), and then calculated pooled effect estimates (SAS MIANALYSE procedure). Further details are available in Table S1, Supplementary Materials. This method allowed us to assess again the association between breastfeeding duration and infections up to 2 years on a sample of 11,238 infants (Figure 1). The model used for these analyses was the same as in our main analysis.

All analyses were carried out with SAS software version 9.4 (SAS Institute, Cary, NC, USA). Statistical significance was defined as *p* < 0.05.

## 3. Results

The comparison between selected families and their non-selected ELFE counterparts is available in Table S2, Supplementary Materials. Briefly, selected mothers were older, less likely to be single parents, born in France, with a higher education level, and employed and they breastfed longer than non-selected mothers.

The sample’s characteristics according to any breastfeeding duration are described in Table 1.

### 3.1. Hospitalizations from Infectious Diseases

Breastfeeding status considered as a binary variable (ever vs. never) was related neither to the number of events (0, 1, ≥2) nor to the cumulative duration of hospitalizations or infectious causes of hospitalizations, whatever the definition of breastfeeding used (any or predominant) (Table 2).

Compared to never-breastfed infants, infants who were predominantly breastfed for <1 month were at higher risk of being hospitalized once, which justifies our sensitivity analysis excluding early infections. The association remained consistent after the exclusion of infants with early hospitalizations (before the age of 2 months) (Table S3, Supplementary Materials).

Compared to never-breastfed infants, infants who were predominantly breastfed for at least 3 months were at lower risk of long duration (≥4 nights) of hospitalizations. These associations remained consistent after the exclusion of infants with early hospitalizations (before the age of 2 months).

Compared to never-breastfed infants, any breastfed for 1 to <3 months infants were at higher risk of hospitalization from fever and any breastfed for <1 month infants were at higher risk of hospitalization from gastrointestinal infections. The first association disappeared after the exclusion of early hospitalization, whereas the second one remained consistent.

### 3.2. Bronchiolitis Events

Overall, the number of bronchiolitis events was significantly related neither to ever breastfeeding nor to breastfeeding duration. However, ever any breastfeeding tended to be related to a higher risk of 1 or 2 bronchiolitis events but not to frequent bronchiolitis events. In contrast, predominant breastfeeding duration tended to be negatively related to the risk of frequent bronchiolitis events (Table 3). These tendencies remained after the exclusion of early hospitalization events (Table S4, Supplementary Materials).

### 3.3. Otitis Events

Both any and predominant breastfeeding were not related to frequent otitis events (at least 3 in the first 2 years) (Table 3).

### 3.4. Antibiotic Use

Ever breastfeeding was related to a lower risk of frequent antibiotic use (at least 2 times in the first 2 years of life), whatever the definition of breastfeeding used (Table 3).

Compared to no breastfeeding, breastfeeding durations (any and predominant) of at least 3 months were associated with a lower risk of frequent antibiotic use.

All these associations remained after the exclusion of infants with early hospitalizations (Table S4, Supplementary Materials).

### 3.5. Analyses after Multiple Imputations

Compared to never-breastfed infants, predominantly breastfed for <1 month infants were no longer at higher risk of a single hospitalization event (OR (95% CI) = 1.17 (0.96; 1.42)) (Table S5, Supplementary Materials). Any breastfed for <1 month infants were also no longer at higher risk of hospitalization from gastrointestinal infections (1.32 (0.99; 1.75)).

Compared to never-breastfed infants, any breastfed for ≥6 months infants were at lower risk of longer hospitalizations (≥4 nights) (0.71 (0.53; 0.95)).

Other previously highlighted results remained consistent (Tables S5 and S6, Supplementary Materials).

## 4. Discussion

In the ELFE study, predominant breastfeeding for over 3 months was related to lower risk of at least 4 nights of hospitalization up to 2 years, while any breastfeeding for over 3 months was related to higher risk of 1 or 2 bronchiolitis events in the first 2 years of age. Finally, both any and predominant breastfeeding durations were negatively associated with frequency of antibiotic use.

We first examined infectious morbidity through common infectious diseases such as bronchiolitis and otitis. Unfortunately, data on diarrhea occurrence were not collected. Contrary to a previous meta-analysis conducted in industrialized countries [12], a lower risk of otitis has not been related to breastfeeding duration. A possible explanation may be the restrictive classification (<3 or ≥3 events) applied in the ELFE questionnaires, not allowing the distinguishing of absence of event from low frequency of events (1 or 2). Consistent with an Italian cohort specifically designed to study respiratory infections [13], we found that, in the main analyses, long duration of predominant breastfeeding (at least 6 months) was associated with a lower risk of frequent bronchiolitis events. Similarly, in the Generation R population-based study, exclusive breastfeeding was related to a lower risk of low respiratory tract infections and, to a lesser extent, upper respiratory tract infections up to 4 years of age [14].

Infections with higher concern can be approximated with hospitalizations. A recent meta-analysis highlighted a protective effect of breastfeeding against hospitalizations from diarrhea and respiratory infections, including lower respiratory tract infection and pneumonia [7]. More recently, the Norwegian MoBa study highlighted a higher risk of hospitalization up to 18 months among infants breastfed for ≤6 months than among those breastfed for at least 12 months [15], but matched sibling analyses, enabled to account for shared maternal characteristics, showed weaker and non-significant associations. We have not observed such associations within the ELFE cohort. Regarding hospitalizations from diarrhea, the meta-analysis included two studies from Europe, highlighting protective associations between breastfeeding and diarrhea [16,17]. Both studies provided data on infants born in the 1990s. Regarding hospitalizations from respiratory infection, the meta-analysis included two studies from Europe, highlighting protective associations between breastfeeding and respiratory infections [18,19], both published before 1995. As infants from the ELFE were born in 2011, they might differ from infants included in these studies. Moreover, in the present analyses only hospitalizations for bronchiolitis could be considered and not all respiratory infections. The low number of hospitalizations for bronchiolitis in our results might prevent any potential association with breastfeeding to arise.

We are unable to provide a biological explanation for the higher risk of 1 or 2 bronchiolitis events in the first 2 years of life related to any breastfeeding but not to predominant breastfeeding. An additional sensitivity analysis adjusting for family history of allergy (parental and/or sibling history of asthma, eczema, and hay fever) did not modify the results (data not shown). A similar unexpected association was found in an Italian case–control study, with a higher breastfeeding rate among infants hospitalized for bronchiolitis than among their control counterparts [20].

Reverse causation bias is a probable hypothesis for the highlighted higher risk of parental reported hospital admission from infection or gastrointestinal infection related to short breastfeeding duration (<1 month) compared to never breastfeeding. As early adverse health events, including hospital admission, can lead to early breastfeeding cessation, the exclusion of early cases of hospitalization allowed us to control this reverse causation bias but not fully as not all adverse health events lead to hospitalization.

Finally, in the present study, antibiotic use was considered as a proxy for bacterial infections. This indicator was strongly related to breastfeeding, a longer duration being related to a lower use of antibiotics up to 2 years of age. Similar results were found in a Czech cross-sectional survey, with lower risk of early exposure to antibiotics among breastfed infants [21]. Likewise, in a Finnish cohort, 1-year breastfed infants were less likely to have been provided with antibiotics during the first year of life than their non-breastfed counterparts [22], and a negative association between breastfeeding duration and antibiotic use was found in a cross-sectional anthropometric and questionnaire study [23]. However, we cannot exclude that health-seeking behaviors could be different among breastfeeding parents and non-breastfeeding parents, leading to the differential use of antibiotics. Moreover, as both breastfeeding and antibiotic use could influence the infant’s microbiome [22], microbiome was suggested as a potential mechanism in the association between breastfeeding and lower rates of infections from hospitalization. It would be of great interest to examine these potential mechanisms from stool samples collected in the first months of life.

The ELFE study is a recent nationwide birth cohort aimed at assessing the development of healthy-born children from birth to adulthood from a broad and interdisciplinary point of view. The prospective design limits recall bias for both exposure and outcomes assessment. However, we have to acknowledge the inability to consider exclusive breastfeeding in the present study according to the WHO definition, because the use of water, water-based drinks, and fruit juice in the 0–2-month period were not collected in the ELFE study. While the study may lack specificity when assessing particular outcomes (e.g., antibiotic types), the strength of our approach is the use of complementary indicators of infectious morbidity, with the occurrence of common infectious diseases, hospitalizations, and antibiotic use. Hospitalizations could reflect the most severe cases and allowed for controlling bias due to parental reporting. It is interesting to note that breastfeeding was more related to the duration of hospitalization than to the number of events. When matching with the national health system database will be available, it would be of great interest to conduct similar analyses based on medical care use rather than parental report. The use of antibiotics would be more specific for bacterial infections, but it remains difficult to distinguish a lower need for antibiotics from the reluctance to such use. The very large sample and the collection of detailed socio-demographic or economic data ensure good statistical power and favor control for potential confounders. Exclusion rates due to missing data for these analyses were high, but multiple imputations did not change the results.

## 5. Conclusions

Even in the context of a high-income country with short breastfeeding duration, we highlighted a lower risk of infectious morbidity related to breastfeeding duration, especially for duration of hospitalization and antibiotic use. The strong association highlighted for antibiotic use would be of great interest in the context of rising concerns regarding antibiotic resistance.

## Figures and Tables

**Table 1:** Included families’ characteristics according to any breastfeeding duration (*n* = 10,349).

Family Characteristics		Breastfeeding Duration				
		Never (*n* = 2489)	<1 Month (*n* = 1704)	1 to <3 Months (*n* = 1629)	3 to <6 Months (*n* = 1964)	≥6 Months (*n* = 2563)
Maternal age at birth (years)		30.4 (4.9)	30.0 (5.0)	30.4 (4.5)	30.9 (4.3)	31.6 (4.6)
Maternal place of birth (France)		96.6% (2405)	94.5% (1611)	92.4% (1505)	90.3% (1774)	82.7% (2120)
Pre-pregnancy body mass index (kg/m²)		23.9 (5.2)	23.8 (4.9)	23.4 (4.5)	22.8 (4.1)	23.0 (4.3)
Education level						
	Below secondary school	7.2% (178)	5.4% (92)	3.8% (62)	3.6% (71)	5.1% (131)
	Secondary school	17.2% (428)	15.8% (269)	9.7% (158)	7.3% (143)	7.9% (202)
	High school	21.6% (537)	21.4% (365)	18.4% (300)	15.1% (297)	13.7% (352)
	2-year university degree	24.5% (611)	25.5% (434)	26.2% (427)	23.7% (465)	21.2% (543)
	3-year university degree	15.8% (394)	18.2% (310)	19.6% (320)	22.3% (437)	22.8% (584)
	5-year university degree or higher	13.7% (341)	13.7% (234)	22.2% (362)	28.1% (551)	29.3% (751)
Employed before pregnancy		77.9% (1938)	75% (1278)	79.7% (1299)	80.2% (1576)	71.4% (1829)
Traditional household composition		87.9% (2188)	87.9% (1498)	91.3% (1487)	91.6% (1799)	90.2% (2311)
Household monthly income (€)		3379 (3171)	3276 (2750)	3665 (4608)	3738 (3671)	3506 (2663)
Smoking status during pregnancy						
	Never smoker	51.9% (1292)	50.1% (853)	57.5% (936)	60.7% (1193)	66.1% (1694)
	Only before pregnancy	24% (598)	27.4% (467)	25.9% (422)	25.1% (492)	23.6% (604)
	Only in early pregnancy	3.5% (88)	4.7% (80)	3.4% (56)	3.8% (74)	2.9% (75)
	Throughout pregnancy	20.5% (511)	17.8% (304)	13.2% (215)	10.4% (205)	7.4% (190)
Caesarean section		19.2% (478)	18% (307)	18% (294)	15.7% (309)	15.2% (389)
Gestational age (weeks)		39.5 (1.5)	39.7 (1.4)	39.7 (1.4)	39.7 (1.4)	39.7 (1.4)
Boys		48.8% (1215)	49.4% (841)	51.6% (841)	50.1% (984)	48.7% (1249)
First born		42.9% (1069)	50.5% (861)	49.7% (809)	45.7% (897)	37.6% (963)
Age at first attendance at a shared childcare facility						
	≤2 months	55.7% (1386)	54.2% (924)	48.4% (788)	46.4% (912)	61.6% (1580)
	>2 months to 4 months	7.4% (183)	5.8% (99)	8% (130)	3.6% (70)	2.1% (53)
	>4 months to 6 months	18.8% (469)	20.4% (347)	26.3% (429)	24.3% (477)	11.2% (286)
	>6 months to 12 months	9.1% (227)	9.2% (157)	8.2% (133)	13.7% (269)	10.7% (273)
	Never attended in the first year	9% (224)	10.4% (177)	9.1% (149)	12% (236)	14.5% (371)
% (n) or mean (± SD)						

**Table 2:** Association between breastfeeding and parent reports of hospitalizations from infection: multivariate analyses (*n* = 10,349).

Breastfeeding Status and Duration		Parental Report of Hospitalizations from Infection											
		Number of Events (Ref = None)			Total Duration (Ref = Never)			Causes					
		1	≥2	*p*	1–3 Nights	≥4 Nights	*p*	Fever	*p*	Gastroint. Inf.	*p*	Bronchiolitis	*p*
Number of infants in each group		842	413		470	429		282		397		475	
Any breastfeeding status				0.52			0.33		0.11		0.40		0.98
	Never	1 (Ref)	1 (Ref)		1 (Ref)	1 (Ref)		1 (Ref)		1 (Ref)		1 (Ref)	
	Ever	1.10 (0.93; 1.31)	1.04 (0.82; 1.32)		1.16 (0.92; 1.46)	0.92 (0.73; 1.16)		1.28 (0.95; 1.74)		1.11 (0.87; 1.42)		1.00 (0.80; 1.24)	
Any breastfeeding duration				0.29			0.25		0.17		0.08		0.10
	Never	1 (Ref)	1 (Ref)		1 (Ref)	1 (Ref)		1 (Ref)		1 (Ref)		1 (Ref)	
	<1 month	1.13 (0.90; 1.42)	1.20 (0.89; 1.62)		1.21 (0.90; 1.63)	1.02 (0.76; 1.38)		1.38 (0.94; 2.02)		1.42 (1.05; 1.91)		0.92 (0.68; 1.24)	
	1 to <3 months	1.22 (0.97; 1.53)	1.15 (0.84; 1.58)		1.17 (0.86; 1.60)	1.08 (0.79; 1.46)		1.55 (1.06; 2.28)		0.91 (0.64; 1.29)		1.24 (0.93; 1.65)	
	3 to <6 months	1.13 (0.91; 1.42)	0.94 (0.68; 1.29)		1.28 (0.96; 1.71)	0.85 (0.63; 1.17)		1.13 (0.76; 1.68)		1.03 (0.75; 1.43)		1.07 (0.80; 1.42)	
	≥6 months	0.96 (0.77; 1.20)	0.89 (0.65; 1.21)		1.00 (0.74; 1.34)	0.77 (0.57; 1.04)		1.13 (0.78; 1.65)		1.04 (0.76; 1.42)		0.82 (0.62; 1.10)	
Number of infants in each group		839	413		468	428		281		395		474	
Predominant breastfeeding status				0.42			0.27		0.65		0.73		0.59
	Never	1 (Ref)	1 (Ref)		1 (Ref)	1 (Ref)		1 (Ref)		1 (Ref)		1 (Ref)	
	Ever	1.10 (0.94; 1.29)	0.96 (0.77; 1.19)		1.12 (0.91; 1.39)	0.88 (0.72; 1.09)		1.06 (0.82; 1.39)		1.04 (0.83; 1.30)		1.06 (0.86; 1.30)	
Predominant breastfeeding duration				0.24			0.08		0.25		0.14		0.24
	Never	1 (Ref)	1 (Ref)		1 (Ref)	1 (Ref)		1 (Ref)		1 (Ref)		1 (Ref)	
	<1 month	1.24 (1.01; 1.52)	1.09 (0.82; 1.44)		1.27 (0.97; 1.65)	1.07 (0.81; 1.40)		1.13 (0.80; 1.59)		1.28 (0.97; 1.68)		1.25 (0.96; 1.62)	
	1 to <3 months	1.13 (0.92; 1.39)	0.99 (0.74; 1.32)		1.05 (0.79; 1.38)	1.00 (0.76; 1.31)		1.28 (0.92; 1.79)		1.02 (0.76; 1.37)		1.04 (0.79; 1.37)	
	3 to <6 months	1.02 (0.82; 1.27)	0.79 (0.57; 1.08)		1.08 (0.81; 1.44)	0.67 (0.49; 0.93)		0.85 (0.58; 1.24)		0.84 (0.61; 1.17)		1.00 (0.75; 1.32)	
	≥6 months	0.90 (0.68; 1.19)	0.92 (0.63; 1.34)		1.05 (0.74; 1.50)	0.67 (0.45; 1.00)		0.88 (0.56; 1.40)		0.89 (0.60; 1.34)		0.81 [0.56; 1.18)	

OR (CI 95%) multinomial logistic regressions adjusted for maternal age at first child, education level, employment status, smoking status during pregnancy and pre-pregnancy BMI, household monthly income per consumption unit, household composition, caesarean section and infant’s sex, gestational age, birth order, and age at first attendance at a shared childcare facility, recruitment wave, maternity unit size and level, and mother’s region of residence. Analyses were performed separately for each breastfeeding definition.

**Table 3:** Association between breastfeeding and parent reports of bronchiolitis events, otitis events, and antibiotic use: multivariate analyses (*n* = 10,349).

Breastfeeding Status and Duration		Parental Report								
		Bronchiolitis Events (Ref = None)			Otitis Events (Ref ≤ 3)		Antibiotic Use (Ref = Never)			
		1 or 2	≥3	*p*	≥3	*p*	Once	2 or 3 Times	>3 Times	*p*
Number of infants in each group		6340	1264		2606		1944	1860	4411	
Any breastfeeding status				0.17		0.32				0.02
	Never	1 (Ref)	1 (Ref)		1 (Ref)		1 (Ref)	1 (Ref)	1 (Ref)	
	Ever	1.11 (0.99; 1.24)	1.09 (0.92; 1.28)		1.06 (0.95; 1.18)		0.94 (0.80; 1.09)	0.84 (0.72; 0.98)	0.83 (0.73; 0.94)	
Any breastfeeding duration				0.05		0.37				0.00
	Never	1 (Ref)	1 (Ref)		1 (Ref)		1 (Ref)	1 (Ref)	1 (Ref)	
	<1 month	1.07 (0.93; 1.24)	1.19 (0.96; 1.48)		1.08 (0.94; 1.25)		0.99 (0.80; 1.22)	1.02 (0.83; 1.26)	1.09 (0.92; 1.30)	
	1 to <3 months	1.09 (0.94; 1.26)	1.08 (0.87; 1.35)		1.12 (0.97; 1.29)		1.04 (0.84; 1.28)	0.95 (0.77; 1.18)	0.98 (0.82; 1.17)	
	3 to <6 months	1.15 (1.00; 1.33)	1.20 (0.97; 1.48)		1.06 (0.92; 1.22)		1.00 (0.82; 1.22)	0.78 (0.64; 0.95)	0.79 (0.67; 0.94)	
	≥6 months	1.12 (0.98; 1.29)	0.90 (0.73; 1.12)		0.98 (0.86; 1.13)		0.79 (0.66; 0.95)	0.71 (0.59; 0.85)	0.61 (0.52; 0.71)	
Number of infants in each group		6335	1263		2605		1940	1857	4410	
Predominant breastfeeding status				0.30		0.92				0.00
	Never	1 (Ref)	1 (Ref)		1 (Ref)		1 (Ref)	1 (Ref)	1 (Ref)	
	Ever	1.08 (0.98; 1.19)	1.04 (0.90; 1.21)		0.99 (0.90; 1.10)		0.96 (0.83; 1.10)	0.84 (0.73; 0.96)	0.79 (0.71; 0.89)	
Predominant breastfeeding duration				0.06		0.98				0.00
	Never	1 (Ref)	1 (Ref)		1 (Ref)		1 (Ref)	1 (Ref)	1 (Ref)	
	<1 month	1.09 (0.95; 1.24)	1.19 (0.98; 1.45)		1.02 (0.89; 1.16)		1.11 (0.92; 1.33)	1.05 (0.87; 1.26)	0.97 (0.83; 1.14)	
	1 to <3 months	1.13 (0.99; 1.30)	1.08 (0.89; 1.32)		1.01 (0.88; 1.15)		0.95 (0.79; 1.14)	0.82 (0.68; 0.99)	0.87 (0.74; 1.01)	
	3 to <6 months	1.10 (0.96; 1.270)	0.99 (0.81; 1.22)		0.97 (0.85; 1.11)		0.88 (0.73; 1.06)	0.71 (0.59; 0.85)	0.65 (0.56; 0.76)	
	≥6 months	0.94 (0.80; 1.110)	0.77 (0.59; 1.00)		0.97 (0.82; 1.15)		0.89 (0.71; 1.10)	0.75 (0.60; 0.93)	0.62 (0.51; 0.75)	

OR (CI 95%) multinomial logistic regressions adjusted for maternal age at first child, education level, employment status, smoking status during pregnancy and pre-pregnancy BMI, household monthly income per consumption unit, household composition, caesarean section and infant’s sex, gestational age, birth order, and age at first attendance at a shared childcare facility, recruitment wave, maternity unit size and level, and mother’s region of residence. Analyses were performed separately for each breastfeeding definition.

## Supplementary Materials

The following are available online at https://www.mdpi.com/2072-6643/11/7/1607/s1. Table S1: Details regarding variables used for the multiple imputations, Table S2: Comparison of included and excluded families (Chi-squared and Student *t*-tests, Table S3: Multivariate adjusted analyses assessing breastfeeding and parent reports of hospitalizations from infection among infants without hospitalization events before 2 months of age (*n* = 9,703), Table S4: Multivariate adjusted analyses assessing breastfeeding and parent reports of bronchiolitis events, otitis events, and antibiotic use among infants without hospitalization events before 2 months of age (*n* = 9,703), Table S5: Multivariate adjusted analyses assessing breastfeeding and parent reports of hospitalizations from infection using multiple imputations to deal with missing measurements on familial or health characteristics (*n* = 11,238), Table S6: Multivariate adjusted analyses assessing breastfeeding and parent reports of bronchiolitis events, otitis events, and antibiotic use using multiple imputations to deal with missing measurements on familial or health characteristics (*n* = 11,238).
