# Supplementary material for: Breastfeeding Status and Duration and Infections, Hospitalizations for Infections, and Antibiotic Use in the First Two Years of Life in the ELFE Cohort
Source: Nutrients. 2019 Jul 15;11(7):1607. doi: 10.3390/nu11071607 (PMC6682893; doi:10.3390/nu11071607)
Supplement: Supplementary file 1 [file nutrients-11-01607-s001.pdf]

Table S1: Details regarding variables used for the multiple imputations

Used variable	Type of variable	Model used to predict missing data	Missing values
Hospital admission	Binary	Logistic regression	0%
Hospital admission from infections	Binary	Logistic regression	0%
Hospital admission from fever	Binary	Logistic regression	0%
Hospital admission from diarrhea	Binary	Logistic regression	0%
Hospital admission from bronchiolitis	Binary	Logistic regression	0%
Hospital admission events	Continuous	Linear regression	0%
Total hospital admission duration	Continuous	Linear regression	0%
Bronchiolitis events	Ordinal (3 categories)	Multinomial regression	0%
Otitis events	Ordinal (3 categories)	Multinomial regression	0%
Food allergy in the first 2 years of life	Binary	Logistic regression	0%
Hospital admission in the first 2 months of life	Binary	Logistic regression	0%
Antibiotic use events	Ordinal (4 categories)	Multinomial regression	0%
Any breastfeeding duration	Continuous	Linear regression	0%
Household composition	Categorical (3 categories)	Multinomial regression	0%
Maternal age at birth	Continuous	Linear regression	0%
Birth order	Continuous	Linear regression	0%
Employement status before pregnancy	Categorical (3 categories)	Multinomial regression	0%
Mother's region of residence	Categorical (9 categories)	Multinomial regression	0%
Maternity unity level	Ordinal (5 categories)	Multinomial regression	0%
Wave of recruitment	Ordinal (4 categories)	Multinomial regression	0%
Educational level	Continuous	Linear regression	0%
Maternal place of birth	Binary	Logistic regression	0%
Predominant breastfeeding duration	Continuous	Linear regression	0%
Gestational age	Continuous	Linear regression	1%
Caesarean section	Categorical (3 categories)	Multinomial regression	1%
Sex	Binary	Logistic regression	1%
Milk feeding type at 2 months	Ordinal (9 categories)	Logistic regression	1%
Maternal smoking during pregnancy	Categorical (4 categories)	Multinomial regression	1%
Maternal BMI	Continuous	Linear regression	1%
Monthly Family income	Continuous	Linear regression	1%
Age at first attendance to shared childcare facility	Ordinal (4 categories)	Logistic regression	4%

Table S2: Comparison of included and excluded families (Chi-squared and Student *t*-tests

	Selected	*Excluded*
**Duration of total any breastfeeding (months)**	4.0± 5.6 (10349)	2.9 ± 4.4 (7652)
**Duration of predominant breastfeeding (months)**	2.2 ± 3.5 (10337)	1.3 ± 2.6 (7790)
**Maternal age at birth (years)**	30.7 ± 4.7 (10349)	29.6 ± 5.4 (7875)
**Place of Birth**		
France	91.0% (9415)	82.6% (5114)
Abroad	9.0% (934)	17.4% (1075)
**Pre-pregnancy BMI (kg/m²)**	23.4 ± 4.7 (10349)	23.6 ± 5.0 (7553)
**Education level**		
Below secondary school	5.2% (534)	12.8% (776)
Secondary school	11.6% (1200)	20.1% (1223)
High school	17.9% (1851)	20.1% (1219)
2-y university degree	24.0% (2480)	18.3% (1113)
3-y university degree	19.8% (2045)	12.9% (781)
5-y university degree or more	21.6% (2239)	15.8% (959)
**Employment status before pregnancy**		
Employed	76.5% (7920)	61.8% (4799)
Unemployed	23.5% (2429)	38.2% (2971)
**Household composition**		
Traditional	89.7% (9283)	82.9% (5133)
Single parenthood	2.8% (293)	8.2% (509)
Step family	7.5% (773)	8.9% (553)
**Household monthly income (€)**	3507 ± 3368 (10349)	3129 ± 2319 (5525)
**Smoking status during pregnancy**		

Never smoker	57.7% (5968)	56.5% (4308)
Only before pregnancy	25.0% (2583)	19.6% (1493)
Only in early pregnancy	3.6% (373)	4.5% (344)
Throughout pregnancy	13.8% (1425)	19.4% (1482)
**Caesarean section**		
No	82.8% (8572)	80.0% (6182)
Yes	17.2% (1777)	20.1% (1550)
**Gestational age (weeks)**	39.7 ± 1.4 (10349)	39.4 ± 1.6 (7589)
**Sex**		
Boys	49.6% (5130)	53.73% (4137)
Girl	50.4% (5219)	46.2% (3551)
**Birth order**		
First born	44.4% (4599)	45.3% (3572)
Second born	87.1% (3841)	33.2% (2617)
Third born	13.8% (1427)	13.9% (1093)
Fourth born or more	4.7% (482)	7.6% (596)
**Age at first attendance to shared childcare facility**		
≤ 2 months	5.2% (535)	8.5% (126)
> 2 months to 4 months	19.4% (2008)	31.9% (471)
> 4 months to 6 months	10.2% (1059)	16.6% (246)
> 6 months to 12 months	11.2% (1157)	27.9% (412)
Never attended in the first year	54.0% (5590)	15.1% (223)

% (n) or mean ± sd (n)

All p-values <0.0001

Table S3: Multivariate adjusted analyses assessing breastfeeding and parent reports of hospitalizations from infection among infants without hospitalization events before 2 months of age (n = 9,703)

	Parental-report of hospitalisations from infection											
	**Number of events (ref = None)**			**Total duration (ref = Never)**			**Causes**					
	**1**	**≥ 2**	**p**	**1-3 nights**	**≥ 4 nights**	**p**	**Fever**	**p**	**Gastroint. Inf.**	**p**	**Bronchiolitis**	**p**
**Number of infants in each group**	589	235		311	207		163		325		240	
**Any breastfeeding status**			0.18			0.15		0.22		0.30		0.81
Never	1 [Ref]	1 [Ref]		1 [Ref]	1 [Ref]		1 [Ref]		1 [Ref]		1 [Ref]	
Ever	1.17 [0.95 ; 1.44]	0.86 [0.64 ; 1.16]		1.23 [0.93 ; 1.63]	0.81 [0.59 ; 1.12]		1.28 [0.86 ; 1.9]		1.15 [0.88 ; 1.51]		0.96 [0.71 ; 1.30]	
**Any breastfeeding duration**			0.55			0.55		0.71		0.14		0.03
Never	1 [Ref]	1 [Ref]		1 [Ref]	1 [Ref]		1 [Ref]		1 [Ref]		1 [Ref]	
<1 month	1.16 [0.89 ; 1.52]	0.92 [0.61 ; 1.37]		1.25 [0.87 ; 1.80]	0.79 [0.51 ; 1.23]		1.28 [0.77 ; 2.12]		1.47 [1.05 ; 2.05]		0.69 [0.44 ; 1.09]	
1 to < 3 months	1.28 [0.98 ; 1.68]	0.95 [0.63 ; 1.43]		1.21 [0.83 ; 1.77]	0.96 [0.62 ; 1.47]		1.45 [0.87 ; 2.42]		0.96 [0.66 ; 1.41]		1.29 [0.88 ; 1.90]	
3 to < 6 months	1.23 [0.95 ; 1.61]	0.79 [0.52 ; 1.19]		1.39 [0.97 ; 1.98]	0.80 [0.52 ; 1.24]		1.22 [0.74 ; 2.04]		1.07 [0.74 ; 1.53]		1.16 [0.79 ; 1.71]	
≥ 6 months	1.05 [0.80 ; 1.36]	0.80 [0.53 ; 1.19]		1.09 [0.75 ; 1.57]	0.74 [0.49 ; 1.13]		1.20 [0.74 ; 1.97]		1.09 [0.77 ; 1.55]		0.76 [0.50 ; 1.16]	
**Number of infants in each group**	587	235		310	206		163		323		240	
**Predominant breastfeeding status**			0.21			0.16		0.65		0.75		0.61
Never	1 [Ref]	1 [Ref]		1 [Ref]	1 [Ref]		1 [Ref]		1 [Ref]		1 [Ref]	
Ever	1.13 [0.93 ; 1.36]	0.84 [0.64 ; 1.12]		1.11 [0.86 ; 1.43]	0.77 [0.58 ; 1.04]		1.08 [0.77 ; 1.53]		1.04 [0.81 ; 1.33]		1.08 [0.81 ; 1.43]	
**Predominant breastfeeding duration**			0.62			0.39		0.74		0.27		0.65
Never	1 [Ref]	1 [Ref]		1 [Ref]	1 [Ref]		1 [Ref]		1 [Ref]		1 [Ref]	
<1 month	1.22 [0.96 ; 1.54]	0.89 [0.61 ; 1.30]		1.27 [0.92 ; 1.75]	0.81 [0.54 ; 1.21]		1.22 [0.79 ; 1.89]		1.25 [0.92 ; 1.69]		1.09 [0.75 ; 1.59]	

1 to < 3 months	1.19 [0.94 ; 1.51]	0.85 [0.59 ; 1.25]	1.02 [0.73 ; 1.42]	0.94 [0.63 ; 1.38]	1.16 [0.74 ; 1.82]	1.06 [0.77 ; 1.46]	1.15 [0.80 ; 1.66]
3 to < 6 months	1.03 [0.79 ; 1.33]	0.79 [0.53 ; 1.18]	1.09 [0.77 ; 1.54]	0.61 [0.39 ; 0.95]	0.89 [0.55 ; 1.47]	0.88 [0.62 ; 1.25]	1.12 [0.77 ; 1.65]
≥ 6 months	0.97 [0.69 ; 1.34]	0.82 [0.49 ; 1.36]	1.01 [0.64 ; 1.59]	0.69 [0.40 ; 1.19]	0.95 [0.52 ; 1.74]	0.80 [0.51 ; 1.28]	0.75 [0.43 ; 1.32]

OR [CI 95%] multinomial logistic regressions adjusted for maternal age at first child, education level, employment status, smoking status during pregnancy and pre-pregnancy BMI, household monthly income per consumption unit, household composition, caesarean section and infant's sex, gestational age, birth order and age at first attendance to shared childcare facility, recruitment wave, maternity unit size and level, and mother's region of residence

Analyses were performed separately for each breastfeeding definition

Table S4: Multivariate adjusted analyses assessing breastfeeding and parent reports of bronchiolitis events, otitis events, and antibiotic use among infants without hospitalization events before 2 months of age (n = 9,703)

	Parental-report								
	**Bronchiolitis events (ref = None)**			**Otitis events (ref = < 3)**		**Antibiotic use (ref = never)**			
	**1 – 2**	**≥ 3**	**p**	**≥ 3**	**p**	**Once**	**2 to 3 times**	**> 3 times**	**p**
**Number of infants in each group**	5949	1112		2399		1855	1744	4046	
**Any breastfeeding status**			0.10		0.33				0.06
Never	1 [Ref]	1 [Ref]		1 [Ref]		1 [Ref]	1 [Ref]	1 [Ref]	
Ever	1.13 [1.01 ; 1.26]	1.06 [0.89 ; 1.26]		1.06 [0.94 ; 1.19]		0.94 [0.80 ; 1.10]	0.87 [0.74 ; 1.01]	0.84 [0.74 ; 0.96]	
**Any breastfeeding duration**			0.13		0.60				0.00
Never	1 [Ref]	1 [Ref]		1 [Ref]		1 [Ref]	1 [Ref]	1 [Ref]	
<1 month	1.09 [0.94 ; 1.27]	1.14 [0.90 ; 1.43]		1.10 [0.94 ; 1.28]		0.96 [0.77 ; 1.19]	1.04 [0.84 ; 1.28]	1.09 [0.91 ; 1.30]	
1 to < 3 months	1.10 [0.94 ; 1.28]	1.02 [0.81 ; 1.29]		1.10 [0.94 ; 1.28]		1.04 [0.84 ; 1.29]	0.98 [0.79 ; 1.22]	0.98 [0.81 ; 1.17]	
3 to < 6 months	1.18 [1.02 ; 1.37]	1.18 [0.95 ; 1.48]		1.05 [0.91 ; 1.22]		1.02 [0.84 ; 1.25]	0.80 [0.65 ; 0.99]	0.81 [0.68 ; 0.96]	
≥ 6 months	1.14 [0.99 ; 1.31]	0.92 [0.74 ; 1.15]		1.00 [0.87 ; 1.16]		0.80 [0.66 ; 0.97]	0.73 [0.61 ; 0.89]	0.64 [0.54 ; 0.75]	
**Number of infants in each group**	5945	1111		2398		1851	1742	4045	
**Predominant breastfeeding status**			0.21		0.92				0.00
Never	1 [Ref]	1 [Ref]		1 [Ref]		1 [Ref]	1 [Ref]	1 [Ref]	
Ever	1.09 [0.99 ; 1.21]	1.04 [0.89 ; 1.21]		1.00 [0.90 ; 1.10]		0.95 [0.82 ; 1.10]	0.84 [0.73 ; 0.97]	0.8 [0.71 ; 0.91]	
**Predominant breastfeeding duration**			0.12		0.99				0.00
Never	1 [Ref]	1 [Ref]		1 [Ref]		1 [Ref]	1 [Ref]	1 [Ref]	

<1 month	1.09 [0.95 ; 1.25]	1.16 [0.94 ; 1.42]	1.00 [0.87 ; 1.15]	1.10 [0.90 ; 1.33]	1.05 [0.87 ; 1.27]	0.97 [0.82 ; 1.15]
1 to < 3 months	1.14 [1.00 ; 1.31]	1.06 [0.86 ; 1.31]	0.99 [0.87 ; 1.14]	0.93 [0.77 ; 1.12]	0.82 [0.68 ; 0.99]	0.87 [0.74 ; 1.02]
3 to < 6 months	1.13 [0.98 ; 1.30]	1.03 [0.83 ; 1.27]	0.98 [0.85 ; 1.12]	0.88 [0.73 ; 1.06]	0.71 [0.59 ; 0.86]	0.66 [0.57 ; 0.78]
≥ 6 months	0.96 [0.81 ; 1.13]	0.78 [0.59 ; 1.03]	1.02 [0.86 ; 1.21]	0.89 [0.71 ; 1.12]	0.77 [0.61 ; 0.96]	0.65 [0.54 ; 0.80]

OR [CI 95%] multinomial logistic regressions adjusted for maternal age at first child, education level, employment status, smoking status during pregnancy and pre-pregnancy BMI, household monthly income per consumption unit, household composition, caesarean section and infant's sex, gestational age, birth order and age at first attendance to shared childcare facility, recruitment wave, maternity unit size and level, and mother's region of residence

Analyses were performed separately for each breastfeeding definition

Table S5: Multivariate adjusted analyses assessing breastfeeding and parent reports of hospitalizations from infection using multiple imputations to deal with missing measurements on familial or health characteristics (*n* = 11,238)

	Parental-report of hospitalisations from infection						
	**Number of events (ref = None)**		**Total duration (ref = Never)**		**Causes**		
	**1**	**≥ 2**	**1-3 nights**	**≥ 4 nights**	**Fever**	**Gastroint. Inf.**	**Bronchiolitis**
**Any breastfeeding duration**							
Never	1 [Ref]	1 [Ref]	1 [Ref]	1 [Ref]	1 [Ref]	1 [Ref]	1 [Ref]
<1 month	1.08 [0.87 ; 1.34]	1.09 [0.81 ; 1.45]	1.16 [0.87 ; 1.54]	0.99 [0.74 ; 1.32]	1.29 [0.89 ; 1.86]	1.32 [0.99 ; 1.75]	0.89 [0.66 ; 1.19]
1 to < 3 months	1.16 [0.93 ; 1.44]	1.08 [0.80 ; 1.46]	1.14 [0.85 ; 1.53]	1.03 [0.77 ; 1.38]	1.44 [1.00 ; 2.08]	0.87 [0.63 ; 1.21]	1.17 [0.88 ; 1.54]
3 to < 6 months	1.08 [0.87 ; 1.33]	0.93 [0.69 ; 1.26]	1.26 [0.96 ; 1.66]	0.85 [0.63 ; 1.15]	1.05 [0.72 ; 1.53]	1.00 [0.74 ; 1.36]	1.04 [0.78 ; 1.37]
≥ 6 months	0.89 [0.72 ; 1.10]	0.82 [0.61 ; 1.11]	0.95 [0.72 ; 1.27]	0.71 [0.53 ; 0.95]	1.06 [0.74 ; 1.52]	0.92 [0.68 ; 1.25]	0.80 [0.61 ; 1.06]
**Predominant breastfeeding duration**							
Never	1 [Ref]	1 [Ref]	1 [Ref]	1 [Ref]	1 [Ref]	1 [Ref]	1 [Ref]
<1 month	1.17 [0.96 ; 1.42]	1.01 [0.84 ; 1.43]	1.23 [0.96 ; 1.59]	1.05 [0.81 ; 1.36]	1.17 [0.85 ; 1.62]	1.23 [0.95 ; 1.60]	1.21 [0.94 ; 1.55]
1 to < 3 months	1.10 [0.90 ; 1.34]	0.95 [0.72 ; 1.26]	1.01 [0.78 ; 1.32]	0.97 [0.75 ; 1.27]	1.27 [0.92 ; 1.76]	1.01 [0.76 ; 1.33]	1.01 [0.78 ; 1.32]
3 to < 6 months	0.97 [0.78 ; 1.19]	0.79 [0.59 ; 1.07]	1.07 [0.82 ; 1.41]	0.66 [0.49 ; 0.90]	0.83 [0.57 ; 1.19]	0.80 [0.59 ; 1.10]	0.99 [0.75 ; 1.31]
≥ 6 months	0.90 [0.69 ; 1.17]	0.89 [0.62 ; 1.28]	1.09 [0.78 ; 1.53]	0.66 [0.45 ; 0.96]	0.90 [0.58 ; 1.40]	0.83 [0.56 ; 1.23]	0.86 [0.61 ; 1.23]

OR [CI 95%] multinomial logistic regressions adjusted for maternal age at first child, education level, employment status, smoking status during pregnancy and pre-pregnancy BMI, household monthly income per consumption unit, household composition, caesarean section and infant's sex, gestational age, birth order and age at first attendance to shared childcare facility, recruitment wave, maternity unit size and level, and mother's region of residence

Analyses were performed separately for each breastfeeding definition

Table S6: Multivariate adjusted analyses assessing breastfeeding and parent reports of bronchiolitis events, otitis events, and antibiotic use using multiple imputations to deal with missing measurements on familial or health characteristics (*n* = 11,238)

	Parental-report					
	**Bronchiolitis events (ref = None)**		**Otitis events (ref = < 3)**	**Antibiotic use (ref = never)**		
	**1 – 2**	**≥ 3**	**≥ 3**	**Once**	**2 to 3 times**	**> 3 times**
**Any breastfeeding duration**						
Never	1 [Ref]	1 [Ref]	1 [Ref]	1 [Ref]	1 [Ref]	1 [Ref]
<1 month	1.08 [0.94 ; 1.25]	1.19 [0.97 ; 1.46]	1.09 [0.95 ; 1.26]	1.01 [0.82 ; 1.23]	1.04 [0.86 ; 1.27]	1.11 [0.93 ; 1.31]
1 to < 3 months	1.11 [0.96 ; 1.28]	1.01 [0.89 ; 1.37]	1.11 [0.97 ; 1.28]	1.02 [0.83 ; 1.24]	0.94 [0.77 ; 1.15]	0.95 [0.80 ; 1.13]
3 to < 6 months	1.19 [1.04 ; 1.37]	1.23 [1.00 ; 1.50]	1.09 [0.95 ; 1.24]	0.98 [0.81 ; 1.18]	0.78 [0.64 ; 0.94]	0.80 [0.68 ; 0.94]
≥ 6 months	1.15 [1.01 ; 1.31]	0.96 [0.78 ; 1.17]	1.01 [0.88 ; 1.15]	0.79 [0.66 ; 0.95]	0.70 [0.58 ; 0.83]	0.63 [0.54 ; 0.73]
**Predominant breastfeeding duration**						
Never	1 [Ref]	1 [Ref]	1 [Ref]	1 [Ref]	1 [Ref]	1 [Ref]
<1 month	1.10 [0.97 ; 1.25]	1.18 [0.98 ; 1.43]	1.03 [0.91 ; 1.17]	1.09 [0.91 ; 1.30]	1.08 [0.91 ; 1.29]	0.98 [0.84 ; 1.14]
1 to < 3 months	1.15 [1.01 ; 1.31]	1.13 [0.93 ; 1.37]	1.01 [0.89 ; 1.15]	0.91 [0.76 ; 1.09]	0.83 [0.69 ; 0.99]	0.85 [0.73 ; 0.99]
3 to < 6 months	1.11 [0.97 ; 1.26]	0.99 [0.81 ; 1.21]	0.98 [0.86 ; 1.11]	0.90 [0.75 ; 1.07]	0.72 [0.60 ; 0.86]	0.67 [0.57 ; 0.78]
≥ 6 months	0.96 [0.82 ; 1.12]	0.83 [0.65 ; 1.07]	0.99 [0.84 ; 1.16]	0.84 [0.68 ; 1.04]	0.71 [0.57 ; 0.88]	0.62 [0.51 ; 0.74]

OR [CI 95%] multinomial logistic regressions adjusted for maternal age at first child, education level, employment status, smoking status during pregnancy and pre-pregnancy BMI, household monthly income per consumption unit, household composition, caesarean section and infant's sex, gestational age, birth order and age at first attendance to shared childcare facility, recruitment wave, maternity unit size and level, and mother's region of residence

Analyses were performed separately for each breastfeeding definition
